# Integration of enzymatic data in *Bacillus subtilis* genome-scale metabolic model improves phenotype predictions and enables in silico design of poly-γ-glutamic acid production strains

**DOI:** 10.1186/s12934-018-1052-2

**Published:** 2019-01-09

**Authors:** Ilaria Massaiu, Lorenzo Pasotti, Nikolaus Sonnenschein, Erlinda Rama, Matteo Cavaletti, Paolo Magni, Cinzia Calvio, Markus J. Herrgård

**Affiliations:** 1 Laboratory of Bioinformatics, Mathematical Modelling and Synthetic Biology, Dep. Electrical, Computer and Biomedical Engineering, University of Pavia, Via Ferrata 5, 27100 Pavia, Italy; 2 Centre for Health Technologies, University of Pavia, Via Ferrata 5, 27100 Pavia, Italy; 3 The Novo Nordisk Foundation Center for Biosustainability, Technical University of Denmark, 2800 Kgs. Lyngby, Denmark; 4 Department of Biology and Biotechnology “Lazzaro Spallanzani”, University of Pavia, Via Ferrata 9, 27100 Pavia, Italy

**Keywords:** Genome-scale metabolic model, Enzymatic data, *Bacillus subtilis*, Constraint-based methods, Poly-γ-glutamic acid

## Abstract

**Background:**

Genome-scale metabolic models (GEMs) allow predicting metabolic phenotypes from limited data on uptake and secretion fluxes by defining the space of all the feasible solutions and excluding physio-chemically and biologically unfeasible behaviors. The integration of additional biological information in genome-scale models, e.g., transcriptomic or proteomic profiles, has the potential to improve phenotype prediction accuracy. This is particularly important for metabolic engineering applications where more accurate model predictions can translate to more reliable model-based strain design.

**Results:**

Here we present a GEM with Enzymatic Constraints using Kinetic and Omics data (GECKO) model of *Bacillus subtilis*, which uses publicly available proteomic data and enzyme kinetic parameters for central carbon (CC) metabolic reactions to constrain the flux solution space. This model allows more accurate prediction of the flux distribution and growth rate of wild-type and single-gene/operon deletion strains compared to a standard genome-scale metabolic model. The flux prediction error decreased by 43% and 36% for wild-type and mutants respectively. The model additionally increased the number of correctly predicted essential genes in CC pathways by 2.5-fold and significantly decreased flux variability in more than 80% of the reactions with variable flux. Finally, the model was used to find new gene deletion targets to optimize the flux toward the biosynthesis of poly-γ-glutamic acid (γ-PGA) polymer in engineered *B. subtilis*. We implemented the single-reaction deletion targets identified by the model experimentally and showed that the new strains have a twofold higher γ-PGA concentration and production rate compared to the ancestral strain.

**Conclusions:**

This work confirms that integration of enzyme constraints is a powerful tool to improve existing genome-scale models, and demonstrates the successful use of enzyme-constrained models in *B. subtilis* metabolic engineering. We expect that the new model can be used to guide future metabolic engineering efforts in the important industrial production host *B. subtilis*.

## Background

*Bacillus subtilis* is the model organism for Gram-positive bacteria and one of the best-characterized bacteria. Its genome has been sequenced and the microbiological, molecular and genetic methodologies for its cultivation and manipulation are well established [1]. Recent studies have generated comprehensive absolute proteome [2] and transcriptome datasets under multiple different growth conditions [3–5]. *B. subtilis* grows efficiently with low-cost carbon and nitrogen sources, it is Generally Recognized as Safe (GRAS) by the US Food and Drug Administration (FDA), and is an attractive chassis for synthetic biology applications thanks to its natural competence, easy chromosomal integration, and capability to efficiently secrete products such as enzymes and vitamins even without engineering [6, 7]. Moreover, *B. subtilis* has important applications in agriculture as plant growth promoting bacterium (PGPB) [8, 9]. Finally, this bacterium has the ability to produce a small, resistant and metabolically dormant spore, which allows easy storage and transportation of *B. subtilis* strains.

For further improvement of productivity of endogenous or exogenous target metabolites in *B. subtilis*, genetic modifications to redirect flux and fermentative process optimization are essential. A wide range of studies have been carried out aimed at optimizing *B. subtilis* as a cell factory for industrially relevant bioproducts such as riboflavin [10, 11], subtilisin [12, 13], synthetic xylanases [14], and poly-γ-glutamic acid (γ-PGA) [15, 16]. These studies have demonstrated industrially relevant improvements in production, but they have also shown that the rational identification of target mutations to optimize the metabolism of *B. subtilis* still represents a major challenge necessitating the use of trial-and-error approaches such as random mutagenesis and screening. In order to complement these labor and cost intensive approaches with rational design tools, multiple different versions of *B. subtilis* genome-scale metabolic models (GEMs) have been reconstructed during the past 10 years [17–20]. Some of these GEMs have been successfully used to drive metabolic engineering applications, such as the production of 3-hydroxypropanoic acid [21], riboflavin, cellulases (R,R)-2, -3-butanediol and isobutanol [20].

Although the available *B. subtilis* GEMs have generally been validated against experimentally determined growth rate data on different carbon substrates as well as by systematic gene essentiality analyses, all the existing models have a relatively low prediction accuracy for central carbon metabolic fluxes and key byproduct secretion rates. Since those models are already fairly complete in terms of representation of the majority of metabolic reactions that can take place in *B. subtilis*, it is likely that prediction accuracy could only be improved through the integration of additional information on bacterial metabolism that is not captured by the current GEMs.

Since the GEMs are generally analyzed through constraint-based methods, the predicted growth rate and the production of the target metabolite are mainly limited by the carbon source uptake rate, constrained based on experimental measurements. However, each metabolic flux is highly dependent on the concentration and kinetics of the enzymes catalyzing the reaction. For this reason, the predictions based only on uptake flux constraints may not agree with the experimental behavior. Different approaches for the integration of enzyme concentrations in GEMs have been developed to constrain the solution space and improve phenotypic predictions. FBAwMC [22] was one of the first approaches, based on the flux balance analysis (FBA) method, imposing concentration constraints for enzymes within the crowded cytoplasm to improve the prediction of growth rates of *Escherichia coli* under different growth media, without using the measurements of nutrient uptake rates. Other methods were proposed as an extension of FBAwMC, such as the metabolic modeling with enzyme kinetics (MOMENT) [23], which utilizes the kinetic parameters under the limitations of the total enzyme pool available. Similarly, Nilsson et al. [24] used an extension of FBA to predict the metabolic trade-offs in yeast, in which the sum of fluxes was constrained to the sum of the product of the maximum in vitro activity and the total enzyme mass. An alternative approach integrates quantitative measurements of protein and metabolite levels into GEM, by associating them with metabolic fluxes by using Michaelis Menten-like rate equations [25]. The most recent and promising method, referred to as GECKO (Enzymatic Constraints using Kinetic and Omics data), uses enzymatic data, in the form of protein abundance and turnover number, as a new constraint for each metabolic flux, ensuring that fluxes do not exceed the maximum capacity in a given condition [26]. GECKO was applied to *Saccharomyces cerevisiae* GEM and it was shown to allow more accurate predictions than the corresponding non-enzyme constrained GEM for growth rates under different carbon sources, for the simulation of the critical dilution rate for Crabtree effect, and for the metabolic behavior of a single-gene knockout strain. The GECKO approach is also relatively simple to implement computationally compared to methods that use more complex rate equations.

In this work, we integrate a set of available enzyme constraints (absolute protein levels and turnover numbers) for the reactions of central carbon metabolism into the iYO844 GEM of *B. subtilis* following the principles of GECKO. The aim was to improve the prediction accuracy of central carbon flux distributions and secretion rates of the main metabolic byproducts with the idea that the resulting model would also allow improved strain design. We use experimental secretion and intracellular flux data of wild-type and mutant strains to evaluate the accuracy of the enzyme-constrained model. We finally demonstrate the metabolic engineering potential of the model by designing knockout strategies for improving the production of γ-PGA, a biopolymer with promising features and applications as food, cosmetics and pharmaceutical additive. We show that the GECKO model predicts different optimal knockout strategies than the standard GEM, and that in vivo implementation of the suggested single-reaction knockouts results in a twofold improvement in γ-PGA production (both rate and titer) from glucose.

## Methods

### Data collection

The kinetic data, in the form of *k*_*cat*_ values (s^−1^), for the enzymes in *B. subtilis* central carbon metabolism, were manually collected from BRENDA [27] and SABIO-RK [28] databases, and scientific literature, together with the respective molecular weights (kDa). We focused further curation of the kinetic data on active reactions in glucose minimal medium and aerobic conditions, also relevant for γ-PGA production. When *k*_*cat*_ values are not directly reported for the characterized enzymes, activity may be expressed as specific activity (SA). This value is defined as the number of micromoles of product formed per milligram of enzyme per minute, at a given temperature and pH. Assuming that the enzyme preparation is 100% pure and that the number of subunits is equal to the number of active sites [29], SA values were converted into *k*_*cat*_ values using the molecular weight (MW) of the enzyme according to Eq. 1.

1 $$k_{{cat}} \left[ {\text{s}^{{ - 1}} } \right] = \frac{{SA~\left[ {\upmu \text{mol/mg/min} } \right] \times MW~\left[ {\text{mg}/ \upmu \text{mol}} \right]}}{{60\,\left[\text{{s/min}} \right]}}$$

For 45 selected enzymes of central carbon metabolism and other closely related reactions reported in the iYO844 model [17], we carried out a manual search for *k*_*cat*_ values and specific activities. We considered only the reactions catalyzed by a unique enzyme, except for the citrate synthase (CS) and oxalate decarboxylase (OXADC) reactions, for which two enzymes are associated but they are mainly catalyzed by CitZ [30] and OxdC [31], respectively, with the second enzyme of both reactions (CitA and OxdD) not giving a relevant contribution (reaction, metabolite and enzyme names are consistent with the ones in the iYO844 model [17]). After this initial filtering, the set of enzymes was reduced to 29. Among these enzymes, we found *B. subtilis* specific *k*_*cat*_ values for 10 reactions. When no measurements were available for *B. subtilis*, *k*_*cat*_ values for *E. coli* were retrieved from the collection reported by Davidi et al. [32], resulting in a data collection for 15 enzymes. However, since the preliminary simulation of flux distribution in wild-type strain, obtained by the integration of this set of 15 reaction data, predicted the activation of two new reactions [methylisocitrate lyase (MICITL), and phosphoglycerate dehydrogenase (PGCDr)] closely connected to central carbon metabolism and known to be inactive, we refined the model by adding enzyme constraints for these two reactions (Table 1).

**Table 1:** List of *k*_*cat*_ values and protein quantifications integrated in iYO844 model

Reaction name	Gene name	EC	Equation	*k*_*cat*_ [s^−1^]	[E] [mmol/g_DW_]	Organism of *k*_*cat*_ data	Refs. for *k*_*cat*_ data
PGI	*pgi*	5.3.1.9	g6p → f6p	126	1.55 × 10^−5^	*E. coli*	[59]
TPI	*tpiA*	4.1.1.31	dhap → g3p	150	1.28 × 10^−5^	*E. coli*	[60]
GAPD_NAD	*gapA*	1.2.1.12	g3p + nad +pi → 13dpg + h + nadh	70	5.77 × 10^−5^	*B. subtilis*	[61]
PGK	*pgk*	2.7.2.3	13dpg + adp → 3pg + atp	329	3.60 × 10^−5^	*E. coli*	[62]
PGM	*pgm*	5.4.2.12	3pg → 2pg	765.9	8.85 × 10^−6^	*B. subtilis*	[63]
ENO	*eno*	4.2.1.11	2pg → h2o + pep	130.4	3.17 × 10^−5^	*B. subtilis*	[64]
G6PDH	*zwf*	1.1.1.49	g6p + nadp → 6pgl + h + nadph	174	8.05 × 10^−6^	*E. coli*	[65]
CS	*citZ*	2.3.3.16	accoa + h2o + oaa → cit + coa + h	49	2.51 × 10^−5^	*B. subtilis*	[30]
ICDHy	*icd*	1.1.1.42	icit + nadp → akg + co_2_ + nadph	82	1.10 × 10^−4^	*B. subtilis*	[66]
FUM	*citG*	4.2.1.2	fum + h2o → mal-l	283.3	7.29 × 10^−6^	*E. coli*	[67]
MDH	*mdh*	1.1.1.37	mal-l + nad → h + nadh + oaa	177.1	1.06 × 10^−4^	*B. subtilis*	[68]
PTAr	*pta*	2.3.1.8	accoa + pi → actp + coa	651.6	8.49 × 10^−6^	*B. subtilis*	[69]
LDH_L	*ldh*	1.1.1.27	lac-l + nad → h + nadh + pyr	6416.6	3.60 × 10^−6^	*B. subtilis*	[70]
PGCDr	*serA*	1.1.1.95	3pg + nad → 3php + h + nadh	14.56	1.90 × 10^−5^	*B. subtilis*	[71]
OXADC	*oxdC*	4.1.1.2	h + oxa → co_2_ + for	59	6.21 × 10^−7^	*B. subtilis*	[72]
MICITL	*yqiQ*	4.1.3.30	micit → pyr + succ	19	6.80 × 10^−8^	*E. coli*	[73]
OXGDC	*menD*	4.1.1.71	akg +h → co_2_ + sucsal	0.2	6.80 × 10^−8^	*B. subtilis*	[55]

For each reaction reported in the table, encoding gene, equation, *k*_*cat*_ and concentration of catalyzing enzyme ([E]) are reported. Moreover, the organism for which the *k*_*cat*_ value was measured and the reference for this measurement are specified. The reaction names, with the associated gene names and equations, correspond to the annotations used in the iYO844 model

As for the absolute protein quantifications, the data reported by Goelzer et al. [33] were used. These values are expressed in number of molecules per cell and are obtained from LC/MS^E^ analysis [2]. This dataset covers most of the cytosolic proteins in the *B. subtilis* 168 strain growing under aerobic batch conditions and in minimal media with different carbon sources. In particular, the measurements in minimal medium with glucose were retrieved from this study and converted into units of millimoles per gram of dry cell weight (mmol/g_DW_) by assuming 6.3 × 10^8^ cells per ml per optical density at 600 nm (OD_600_) [2] and 0.48 g_DW_/L per OD_600_ [33]. For each enzyme with known *k*_*cat*_, we used the upper limit of the 95% confidence interval of protein abundance value in order not to over-constrain the model predictions (Table 1). When the protein level quantification of a specific enzyme was not available, we assumed that the protein is present at levels under the detection limit, and the minimum value among all the protein level measurements in the same condition (i.e., 6.8 × 10^−8^ mmol/g_DW_) was used.

### Integration of enzymatic data in the model

The enzymatic data reported in Table 1 were integrated into the iYO844 model, consisting of 1020 reactions, in order to obtain an enzyme-constrained model [26] that can be easily used with standard metabolic engineering design tools as it retains the linear structure of the original model and only adds additional constraints to a subset of model reactions. To implement the enzymatic data integration following the GECKO approach [26], an additional constraint was considered so that the metabolic flux through the j-th reaction (*R*_*j*_), reported in Table 1, does not exceed its maximum capacity (*v*_*max*_), corresponding to the product between the *k*_*cat*_ value (converted to h^−1^) of the enzyme *E*_*j*_ (that catalyzes the j-th reaction) and its abundance [*E*_*j*_], as shown in Eq. 2.

2 $${v}_{j}\le {k}_{cat}^{j}\cdot \left[{E}_{j}\right]\quad for \;\;\;\; j=1\ldots 17$$

Since we considered reactions catalyzed by unique enzymes, the number of enzyme-constrained reactions (17) is equal to the number of enzymes.

In summary, each constrained metabolic reaction *R*_*j*_ includes a pseudo-metabolite representing enzyme usage, which is limited by protein abundance.

Following the GECKO approach [26] to implement the described method, as a first step, the iYO844 model was converted into an irreversible model and the constraint for uptake rate of glucose, the sole carbon source, was removed. Then, the stoichiometric matrix and the upper bound vector of the model were expanded by adding the *k*_*cat*_ values and the known protein abundances (Table 1).

Specific proteomic data for the mutant strains tested in this work were not available. For this reason, the approach shown above was applied under the assumption that enzyme concentrations in wild-type and mutant strains are the same, except for the enzyme associated to the deleted reaction, whose concentration was fixed to zero. An alternative approach was tested for mutant strain simulations, in which only the total amount of enzymes was constrained (Eq. 3), similar to previously proposed approaches [22, 23]:

3 $$\sum _{i}^{17}{MW}_{i}\cdot \left[{E}_{i}\right]\le f\cdot {P}_{total}$$

The kinetic data, in terms of turnover number, were integrated in the stoichiometric matrix (S) as in the standard approach. When using this approach, the total amount of cellular proteins (*P*_*total*_) in the cell was assumed to be 0.55 g/g_DW_, corresponding to the value measured for *E. coli* [34], and the mass fraction of the accounted proteins (*f*) was computed equal to 0.0191, by summing the abundance, expressed as parts per million (ppm), of the 17 considered proteins, retrieved from PaxDB database [35]. All the computations were implemented via MathWorks MATLAB R2012a and run with COBRA toolbox [36] using the Gurobi solver [37].

### Simulations

For the in silico simulation of each mutant strain, the reaction encoded by the knocked-out gene was imposed as inactive, namely with a flux equal to zero. All strains were simulated using both the previously published iYO844 model and the new enzyme-constrained model obtained in this work, hereafter referred to as ec_iYO844. The lower bound of glucose uptake rate was fixed to the specific experimental value for the iYO844 model (− 7.71 mmol/g_DW_/h for wild-type strain), based only on stoichiometric reactions and directionality, whereas it was set to an unlimited value (− 100 mmol/g_DW_/h) for ec_iYO844. The metabolic phenotype of wild-type *B. subtilis* strain was predicted by parsimonious flux balance analysis (pFBA) [38]. In addition, mutant strains were simulated using the minimization of metabolic adjustment (MoMA) method [39], which minimizes the distance between the flux distributions in wild-type and mutant strains.

The range of each flux value supporting 90% of maximum growth rate was evaluated by flux variability analysis (FVA) as previously described [40] for both models, using an unlimited glucose uptake rate value (constrained to − 100 mmol/g_DW_/h). The variability range of each flux (FV) was computed as in Eq. 4.

4 $$FV=maxflux-minflux$$

The pFBA, MoMA and FVA methods were run in Matlab using the available functions in the COBRA Toolbox [36].

### Identification of deletion targets

The gene deletions that are required to optimize the production of γ-PGA were identified by using the iYO844 model and ec_iYO844. The MoMA method was used to find single or multiple gene deletions corresponding to the best trade-off between growth rate and secretion rate of the target metabolite as previously described [41].

Since the *pgs* operon, including the enzyme-encoding genes responsible for γ-PGA production, is not expressed in the laboratory strain modeled in iYO844, we added the production reaction reported in the GEM of *Bacillus licheniformis* [42] (0.77 glu-D + 0.23 glu-L  →  γ-pga). As an alternative approach to optimize γ-PGA production, we considered the flux maximization of three of its known precursors: 2-oxoglutarate (akg), d-glutamate (glu-d) and l-glutamate (glu-l). In this case, the secretion reactions of these precursors were added to the model.

### Evaluation of prediction accuracy

Flux distribution and growth rate data of *B. subtilis* wild-type and single-gene/operon deletion strains, grown under M9 minimal medium with glucose, were retrieved from literature [43, 44]. In these datasets, internal fluxes, measured by ^13^C-labeling experiments, are reported for the main reactions of the central carbon pathway, together with growth, glucose uptake and acetate production rates. For each measured flux the coefficient of variation among the replicates, when available in literature, is typically lower than 10%, with only the reactions of pentose phosphate pathway and malate dehydrogenase (MDH) of the tricarboxcylic acid (TCA) cycle having slightly higher variability (< 38%).

The prediction error was computed for each simulation of a strain grown under a specific condition by the normalized Euclidean distance between the experimental and the respective predicted fluxes [45] (see Eq. 5).

5 $$pred\,error= \frac{||exp\,flux -pred\,flux||}{||exp\,flux||}$$

Furthermore, 95 single-gene deletions, corresponding to genes included in the GEM and experimentally found to be lethal in genome-wide studies [44, 46], were simulated via MoMA with the iYO844 and ec_iYO844 models, and the percentage of correctly predicted essential genes (i.e., yielding a predicted growth rate lower than 0.05 h^−1^) was calculated and compared to experimental data.

### Additional model analyses

In order to evaluate the impact of *k*_*cat*_ variation on prediction accuracy of the enzyme-constrained model, a distribution of prediction errors was computed by simulating ec_iYO844 using 10,000 *k*_*cat*_ sets, randomly extracted without replacement from the list reported in Table 1.

In addition, to investigate the minimum set of reactions, among the ones reported in Table 1, that must be enzyme-constrained to achieve the final accuracy of ec_iYO844, the model was studied by following a stepwise inclusion procedure for the constrained reactions. In particular, unless differently indicated, the prediction errors for wild-type and mutant strains, together with the accuracy of essential genes, were all taken into account as indices to evaluate the accuracy.

### Strain construction

The strains used in this study are listed in Table 2 with their relevant genotype. Briefly, PB5249 is a spontaneous *swrA*^+^ derivative of the laboratory strain JH642 [47]. PB5383 is a γ-PGA producer thanks to the double mutation *swrA*^*+*^* degU32*(Hy). PB5642 and PB5643 were obtained by transforming the genomic DNA of strains GP791 (Δ*sucC*-*sucD*::Tet) and GP1276 (Δ*odhA*-*odhB*::Cat) (Jörg Stülke Lab, Gottingen, Germany), into PB5249 and selecting deletion strains with tetracycline (20 µg/mL) and chloramphenicol (5 µg/mL), respectively. The final γ-PGA producer knockout strains PB5691 and PB5716 were obtained by transferring the *degU32*(Hy) mutation into PB5643 and PB5642, respectively, via transformation of PB5383 genomic DNA and selection with spectinomycin (60 µg/mL). Deletions were confirmed via colony PCR with the primer pairs SucCDForCheck (5′-gattttgcatcgaactgtagac-3′)—TetRRevCheck (5′-gtcgtaaattcgattgtgaa-3′) for Δ*sucCD* and OdhABForCheck (5′-gtagaatcaaattgcaaacagtgg-3′)—CAT-R-50 (5′-gtctgctttcttcattagaatcaatcc-3′) for Δ*odhAB*. The presence of *swrA*^*+*^* degU32*(Hy) mutations could be easily verified via the mucoid phenotype on agar plates and clear zone on skim milk plate assay.

**Table 2:** *B. subtilis* strains used in this study

Strain	Genotype	Refs.
PB5249	*trpC2 pheA1 swrA* ^*+*^	[74]
PB5383	*trpC2 pheA1 swrA*^*+*^* degU32*(Hy) (Sm^r^)	[15]
PB5643	*trpC2 pheA1 swrA*^*+*^* ∆odhAB* (Cat^r^)	This study
PB5642	*trpC2 pheA1 swrA*^*+*^* ∆sucCD* (Tet^r^)	This study
PB5691	*trpC2 pheA1 swrA*^*+*^* degU32*(Hy) (Sm^r^) *∆odhAB* (Cat^r^)	This study
PB5716	*trpC2 pheA1 swrA*^*+*^* degU32*(Hy) (Sm^r^) *∆sucCD* (Tet^r^)	This study

### Growth conditions and fermentation

The PB5383, PB5691 and PB5716 strains from a streaked LB agar plate (tryptone, 10 g/L; yeast extract, 5 g/L; NaCl, 10 g/L; agar, 15 g/L) were grown for 7 h in 2.5 mL of Penassay broth (Difco Antibiotic Medium 3) with 5 g/L glucose in an orbital shaking incubator at 37 °C, 250 rpm; cultures were diluted to an OD_600_ of 0.1 in 20 mL of E medium adapted from Leonard et al. [48] (citric acid, 12 g/L; glucose, 80 g/L; NH_4_Cl, 7 g/L; K_2_HPO_4_, 0.5 g/L; MgSO_4_·7H_2_O, 0.5 g/L, FeCl_3_·6H_2_O, 0.04 g/L; CaCl_2_·2H_2_O, 0.15 g/L; MnSO_4_·H_2_O, 0.104 g/L; tryptophan, 50 µg/mL; phenylalanine, 50 µg/mL) with 40 g/L of l-glutamic acid (EM+) or without l-glutamic acid (EM−) and incubated under the same conditions as above. In fermentation experiments, aliquots (500 µL) were collected for OD_600_ readings and γ-PGA extraction at the following time points: 0, 18, 25, 43, 49, 66 and 74 h. In growth experiments, pre-cultures in EM− were grown for 16 h and then diluted to OD_600_ 0.1; finally, OD_600_ was monitored for 9 h during the exponential growth phase, thereby enabling the calculation of growth rate as previously described [49].

### γ-PGA recovery and quantification

Each culture sample was centrifuged (16,000 rpm, 20 min, 4 °C) and the cell pellet was discarded. The supernatant was precipitated with three volumes of cold methanol and kept at − 20 °C for at least 12 h. For γ-PGA quantification, each sample was centrifuged (14,000 rpm, 15 min, 4 °C) and methanol was discarded; γ-PGA pellets were dried in a vacuum concentrator and then dissolved in deionized water. The concentration of γ-PGA was measured by spectrophotometer at 216 nm in a quartz cuvette, as previously reported [50]. Absorbance measurements were carried out via the NanoPhotometer UV/Vis spectrophotometer (Implen, Schatzbogen, München, Germany), using deionized water as blank, and a standard calibration curve with purified γ-PGA (#G1049, Sigma Aldrich, dissolved in deionized water) to compute its concentration in g/L from absorbance values. Measurements were carried out for at least three independent fermentation experiments.

The same aliquots of γ-PGA produced by wild-type and mutant strains were also visually compared by agarose gel electrophoresis followed by methylene blue staining, as previously described [51].

### Statistical tests

Microsoft Excel and Matlab functions were used to carry out statistical tests. Analysis of variance (ANOVA) was used to compare γ-PGA concentration or production rate among the three producer strains (PB5383, PB5691 and PB5716). A priori pairwise orthogonal comparisons were carried out via individual T-tests between PB5383 vs knockout strains and between the two knockout strains.

Wilcoxon signed rank test was used to compare flux variability values for all the model reactions with non-zero FV values. The metabolic subsystems (retrieved from the iYO844 original model) over-represented in the list of reactions with relevant flux variability reduction were found by Fisher’s exact test.

## Results

A new enzyme-constrained GECKO model of *B. subtilis* (ec_iYO844) was constructed by integrating publicly available enzyme kinetic and proteomic data for a set of central carbon and related pathway reactions (Table 1). The prediction performance of ec_iYO844 was compared to that of the original model (iYO844), in terms of ability to capture flux distributions in wild-type and single-gene/operon deletion mutants, and essential genes. The two models were also compared in terms of flux variability analysis, to evaluate if enzyme constraints were able to decrease the variability of fluxes in one or more pathways. Finally, the predicted knockout strategies to improve γ-PGA production were compared between the models and the suggested single-reaction deletions were evaluated in vivo.

### Evaluation of model prediction performance on wild-type strain

**Fig. 1:**
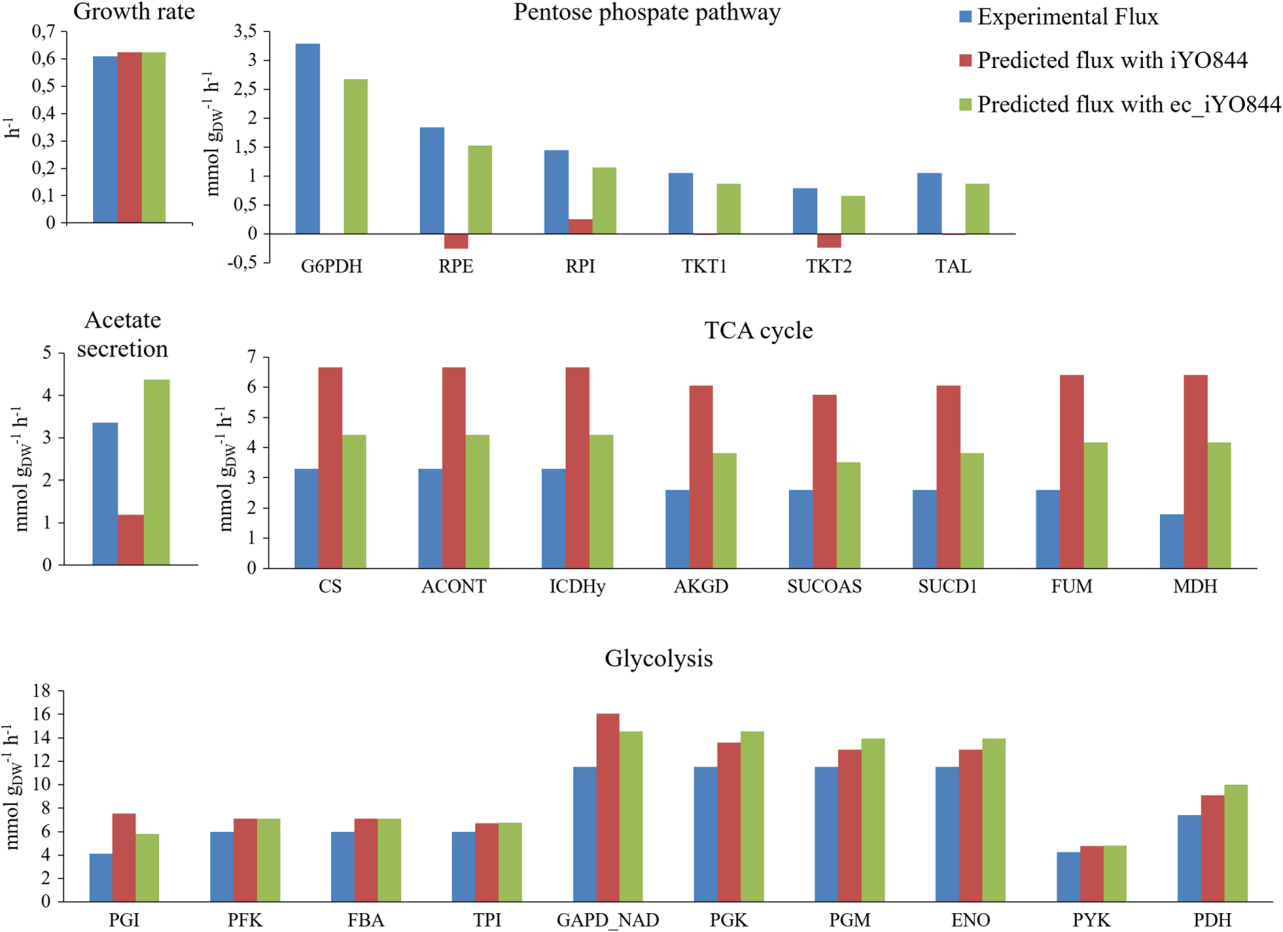
Experimental and predicted fluxes of central carbon reactions for wild-type *B. subtilis*. The predictions of growth rate, acetate secretion rate, fluxes through the reactions of glycolysis, TCA cycle and pentose phosphate pathway for wild-type strain, using iYO844 and ec_iYO844 (red and green bars, respectively), are compared to the experimental measurements (blue bars). Reaction names are reported according to the annotation used in the original model

The comparison between the predicted wild-type flux values and the corresponding experimental fluxes available in the literature [43] (Fig. 1) showed that iYO844 model was able to accurately predict growth rate and fluxes through the glycolytic pathway. However, the pentose phosphate pathway (PPP), TCA cycle and acetate secretion fluxes were not consistent with experimental values. In particular, the flux through the PPP and acetate production were significantly underestimated, while the flux through TCA cycle was overestimated about twofold. A significant improvement was achieved with the integration of enzymatic data (ec_iYO844), including the ability to predict the glucose uptake rate (predicted to be 8.62 mmol/g_DW_/h, consistent with the experimental value of 7.71 mmol/g_DW_/h), and the correction of the inaccurate flux predictions by iYO844 in the three pathways described above, without reducing accuracy of glycolytic flux and growth rate predictions. The overall increase in prediction accuracy using the enzyme-constrained model is confirmed by the 43% decrease of the prediction error, from 0.47 to 0.27.

### Evaluation of model prediction performance on mutant strains

**Fig. 2:**
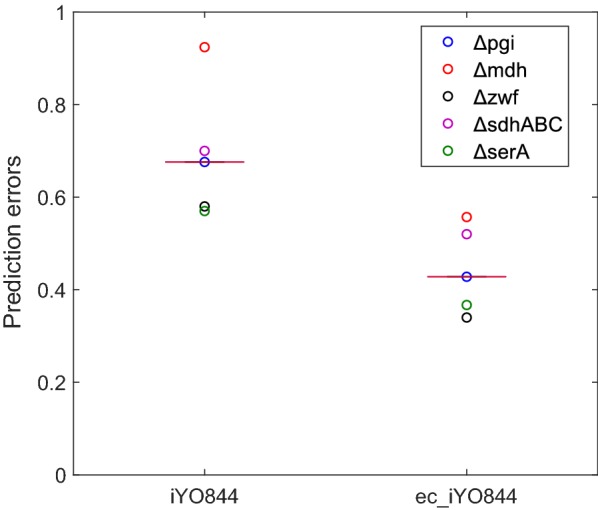
Distribution of prediction errors for *B. subtilis* mutant strains. Circles represent the prediction error distribution, computed with iYO844 and ec_iYO844, for each mutant strain considered in this work and the red lines indicate the median values

We also tested the prediction performance of the ec_iYO844 model under perturbed genetic conditions. We simulated the metabolic behaviors of five single-gene/operon deletion strains: ∆*pgi*, ∆*mdh*, ∆*zwf*, ∆*sdhABC* and ∆*serA*, for which the glucose-6-phosphate isomerase (PGI), MDH, glucose 6-phosphate dehydrogenase (G6PDH), succinate dehydrogenase (SUCD1) and PGCDr reactions are blocked, respectively. The experimental fluxes of four central carbon reactions [PGI, G6PDH, pyruvate kinase (PYK), and CS], growth rate and acetate production rate [44] were compared with the corresponding predicted values, obtained by the two models. Similar to the observations for the wild-type strain, for the mutant strains results show an overall improvement of prediction accuracy when enzymatic data are integrated. Considering the distribution of prediction errors of all mutants, the median value decreases by 36%, from 0.67, using the initial model, to 0.43 (Fig. 2).

**Fig. 3:**
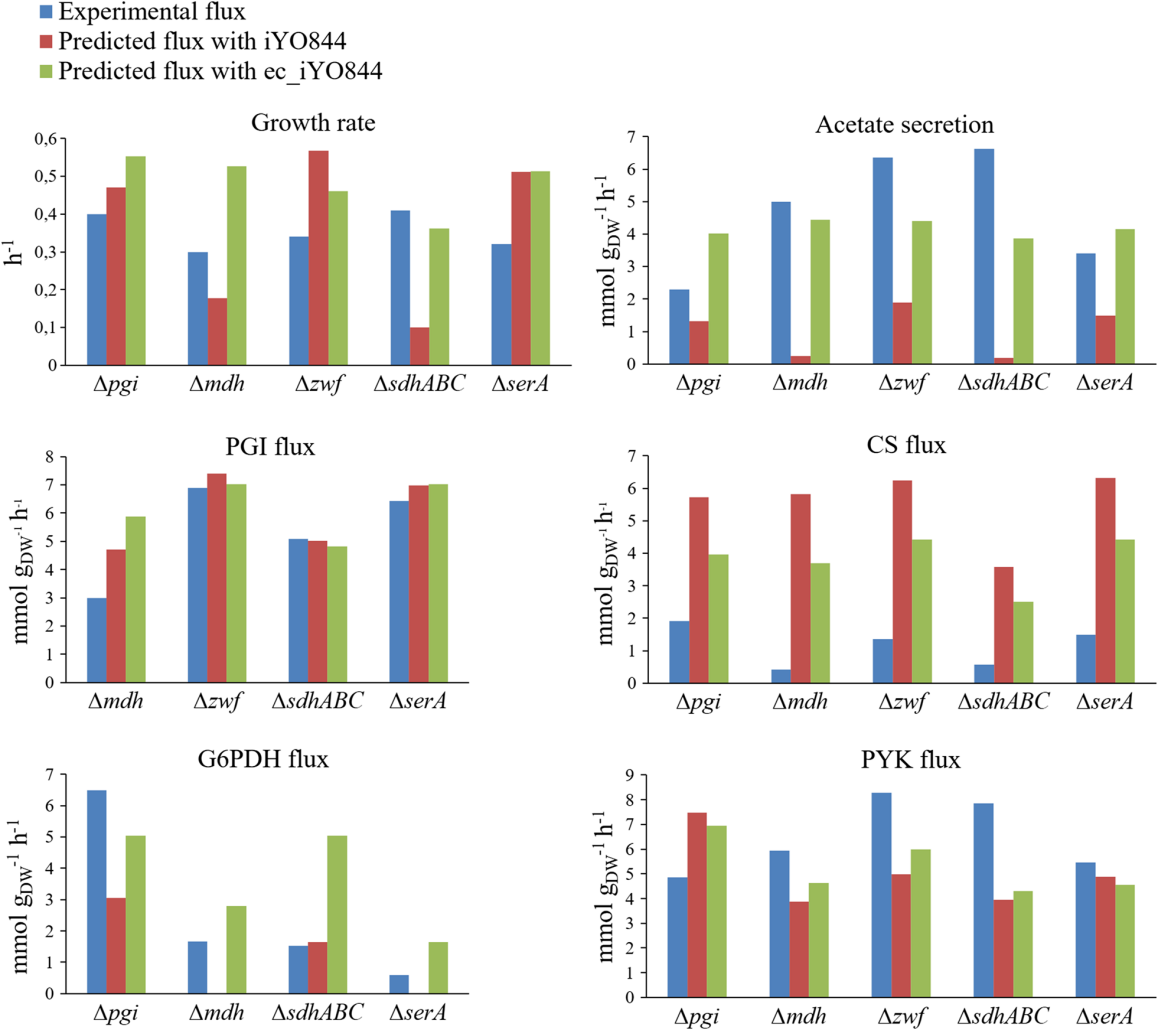
Experimental and predicted fluxes for *B. subtilis* mutant strains. Five different mutants strains (∆*pgi*, ∆*mdh*, ∆*zwf*, ∆*sdhABC* and ∆*serA*, for which the PGI, MDH, G6PDH, SUCD1 and PGCDr reactions are blocked, respectively) were simulated using iYO844 and ec_iYO844 model (red and green bars, respectively). The predictions of growth rate, acetate secretion rate and fluxes through PGI, G6PDH, PYK, CS reactions are compared with the experimental measures (blue bars). Reaction names are reported according to the annotation used in the original model

While the mutant strains prediction error showed a considerable decrease using ec_iYO844 compared with iYO844, not all the individual flux predictions were similarly improved (Fig. 3). This was in contrast with the predictions obtained for wild-type strain for which we observed a clear improvement for all the individual reactions (Fig. 1). As it was also observed in wild-type flux prediction, ec_iYO844 showed a relevant improvement of accuracy for acetate secretion rate: iYO844 systematically predicted values lower than those measured for each strain, especially for ∆*mdh* and ∆*sdhABC* strains, in which the MDH and SUCD1 reaction is blocked, respectively, with predicted acetate flux value close to zero. However, upon integration of enzymatic data in the model, the predicted acetate secretion rates became more consistent with the experimental values, although the model was still unable to predict the highest rates measured (∆*zwf* and ∆*sdhABC* strains). An overall improvement was also observed in the flux value prediction of the G6PDH reaction (the first one of PPP, encoded by *zwf*), in three cases (∆*pgi*, ∆*mdh* and ∆*serA*), with experimental flux ranging from 0.6 to 6.5 mmol/g_DW_/h), two of which corresponding to a flux value wrongly predicted as zero by iYO844. Conversely, in the ∆*sdhABC* strain, ec_iYO844 captured the G6PDH flux value less accurately than iYO844 indicating that the absolute protein levels in this strain may differ from those in the wild-type strain, which were used in the simulation.

As expected, with the model developed in this work, the fluxes through the CS reaction in the TCA cycle decreased in every mutant strain, resulting into higher consistency with experimental values, due to the flux constraint of 4.4 mmol/g_DW_/h imposed by enzymatic information (derived from Table 1).

Taken together, the results showed that new model is also superior in terms of mutant strain flux predictions, although with a percent improvement in prediction accuracy lower than what was observed for the wild-type strain. The strong assumption that proteomic profile is the same in wild-type (for which proteomic data was available) and single-gene/operon mutants may be partly responsible of the less accurate predictions obtained for mutant strains. For the enzyme-constrained model, we also used an alternative approach, similar to the MOMENT method [23], in which the *k*_*cat*_ values for the 17 reactions were integrated and only the total amount of enzymes (g/g_DW_) was constrained (kc_iYO844 model). The results showed that this method has lower prediction error (0.55 median value) than iYO844 (0.67 median value), but, also in this context, the predictions obtained by ec_iYO844 are the most accurate (0.43 median value). The three models (iYO844, ec_iYO844 and kc_iYO844) were also solved via pFBA instead of MoMA, but conclusions do not change and the resulting prediction errors with pFBA are always higher than the respective ones with MoMA (data not shown).

The new model was also evaluated by gene essentiality analysis (Table 3), a step commonly performed for validation of new GEMs. Considering 11 single-gene knockout strains in central carbon metabolism genes from a list of 95 experimentally-tested essential genes, the no-growth phenotype was correctly predicted by iYO844 only for two strains (∆*pgk* and ∆*tkt*). On the other hand, the model developed was able to predict the essentiality of three additional central carbon metabolism genes (*pfkA*, *eno* and *pgm*), for a total of 5 out of 11 correctly predicted knockout phenotypes. No difference was observed in the accuracy of the prediction for the remaining essential genes belonging to other pathways: 67 of them were correctly predicted as essential by both models.

**Table 3:** Gene essentiality analysis

Essential genes			
	CC	Others	Total
Experimental data	11	84	95
Predicted with iYO844	2	67	69
Predicted with ec_iYO844	5	67	72

Total number of essential genes reported in literature [44, 46], and those predicted using iYO844 and ec_iYO844. The number of genes coding for enzymes of the central carbon (CC) pathway are specified in the first column

### Impact of the knowledge of enzymatic data

The impact of the specific types of enzymatic data used to develop the enzyme-constrained model on model performance was analyzed in two tests.

First, among the 17 enzyme-constrained reactions, we identified triose-phosphate isomerase (TPI), glyceraldehyde-3-phosphate dehydrogenase (GAPD_NAD), CS and PGCDr as the minimum set required to achieve the final prediction performance of the model developed, namely a prediction error equal to 0.27 for the wild-type strain and to 0.43 for the mutants, and 76% of accuracy for central carbon gene essentiality. This result, however, is expected to depend on the study on which the model is applied, with prediction accuracy increasing with the number of enzyme-constrained reactions. This outcome is also suggested by our gene essentiality analysis, in which a significant improvement was observed for central carbon pathway but not in other pathways that were not subjected to enzymatic constraints.

By considering the prediction error for wild-type and mutant strains as the only performance measure, the GAPD_NAD and CS constraints were sufficient to reach the maximum predictive power. The importance of these constraints could be due to the key role of glyceraldehyde 3-phosphate (converted into 3-phospho-d-glyceroyl-phosphate via the GAPD_NAD reaction) as a central node between glycolysis and pentose phosphate pathway, and the key role of citrate synthesis (from oxaloacetate and acetyl-CoA via the CS reaction) as central node between glycolysis and the entering of TCA cycle. To better elucidate the role of these two reactions, we performed three simulations in which the GAPD_NAD and CS reactions were individually or jointly constrained as the sole constraint(s) of the ec_iYO844 model. The model reached its lowest error only by constraining both reactions. The individual constraint of GAPD_NAD or CS was not able to fix the PPP flux distribution, while the improvement in the TCA cycle fluxes was observed by individually including the CS constraint.

Conversely, considering the accuracy of gene essentiality analysis, the TPI and PGCDr constraints were needed to correctly predict *pfkA*, *eno* and *pgm* as essential genes, which catalyze the PFK, ENO and PGM reactions, respectively (Fig. 6). To better investigate the role of these two constraints, we performed simulations in which TPI or PGCDr were constrained. Results showed that the two constraints individually contributed to the correct prediction of *pfkA* (TPI constraint), and of *pgm* and *eno* (PGCDr constraint).

**Fig. 4:**
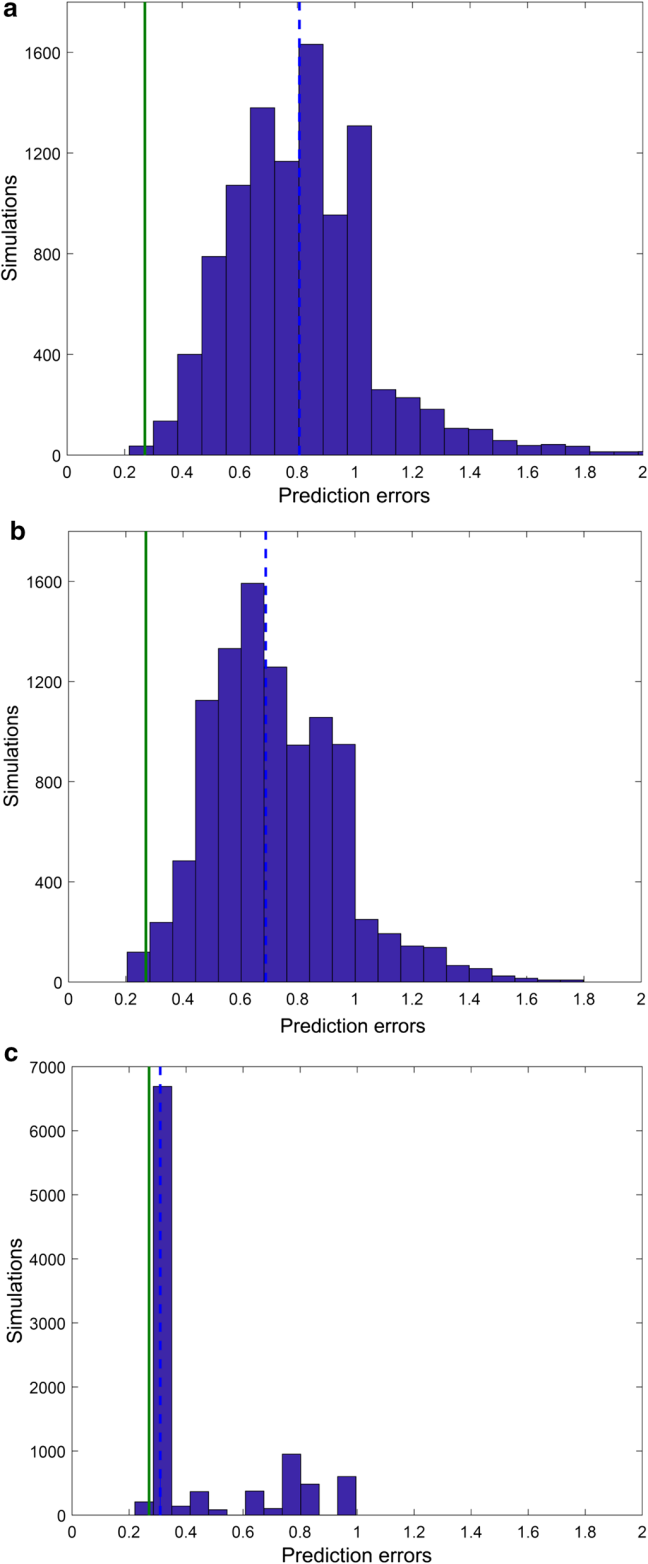
Distribution of predicted errors obtained from enzyme-constrained model with randomized *k*_*cat*_ values on wild-type strain. Bars represent the histograms obtained on 10,000 simulations, dashed line indicates the median value of the prediction errors obtained using randomized *k*_*cat*_ values and solid line represents the prediction error of the ec_iYO844 model. Panels show the results for the *k*_*cat*_ values randomization of all the 17 reactions (**a**), all the reactions except GAPD_NAD and CS (**b**) and only GAPD_NAD and CS (**c**)

Second, to further evaluate the impact of enzymatic constraints, we tested how much model performance depended on specific *k*_*cat*_ values by using randomized *k*_*cat*_ for all the 17 reaction constraints. The results (Fig. 4a) showed that the median value of prediction errors using random *k*_*cat*_ (0.80) is significantly higher than the value obtained by the enzyme-constrained model (0.27, lower than the first percentile of the obtained distribution), and even higher than the one of the model without constraints (0.47). The knowledge of specific *k*_*cat*_ values is therefore essential to obtain accurate predictions. We also tested the effect of *k*_*cat*_ randomization for 15 of the 17 reactions, leaving the GAPD_NAD and CS constraints (found to be essential to reach the final accuracy) to their correct values. The results (Fig. 4b) showed that the distribution of prediction errors is similar to the one obtained above (median = 0.69 and the final prediction error value represented the first percentile). This demonstrates that the randomization of *k*_*cat*_ values has a detrimental effect, even when random constraints are imposed for the reactions not contributing to minimize the prediction error. Finally, we tested the effect of *k*_*cat*_ values randomization only on the GAPD_NAD and CS reactions. The obtained distribution of prediction errors (Fig. 4c) has much lower median (0.31) than the distributions obtained above, and this median is also lower than the error of the model without constraints (0.47). However, the prediction error of ec_iYO844 with all the 17 constraints (0.27) is in the second percentile of this distribution, thereby confirming the importance of setting correct values for the GAPD_NAD and CS constraints, and also that correct values of the constraints for the other reactions contribute to the significant decrease in prediction error.

### Flux variability analysis

**Fig. 5:**
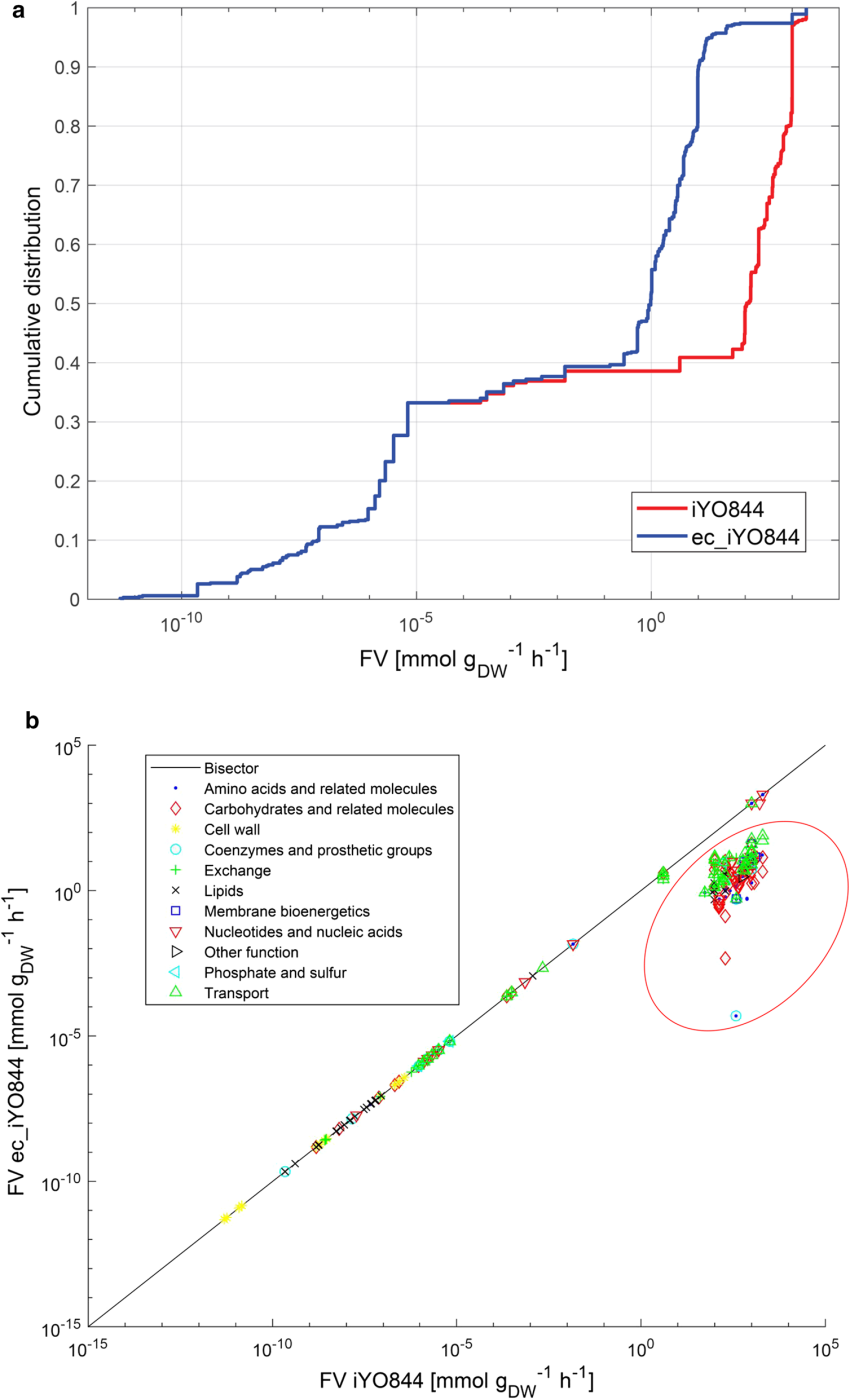
Flux variability analysis on the two models. **a** Cumulative distribution of all the non-zero FV values. **b** Identification of reactions and their respective metabolic subsystem with major variability reduction after the enzymatic data integration (red circle)

To compare the flux solution spaces of the enzyme constrained and non-constrained models, flux variability analysis was carried out, using an unlimited glucose uptake rate value. The cumulative distribution of flux variability (FV) values demonstrated a relevant FV reduction for ec_iYO844 compared to iYO844, with a 120-fold decrease of median FV (Fig. 5a) (p-value < 0.05; Wilcoxon signed rank test). Considering all the non-zero FV values (653 reactions), 81% of all the fluxes showed variability reduction using ec_iYO844, whereas only 11% showed an increase and 8% showed an unchanged variability. The distribution of FV values suggests that the main improvements in variability reduction are in the iYO844 reactions with high FV values. A cluster of 357 reactions showing major variability reduction was considered (Fig. 5b), in order to analyze which pathways were enriched in that set. The 17 enzyme-constrained reactions in ec_iYO844 (Table 1) mainly belong to the "Carbohydrates and related molecules" group, with a minor amount belonging to the "Amino acids and related molecules", "Membrane bioenergetics" and "Coenzymes and prosthetic group" subsystems. As expected, the over-represented metabolic subsystems in the low-FV reaction cluster included the "Carbohydrates and related molecules" group, but also the "Nucleotides and nucleic acids" one (p-value < 0.05; Fisher's exact test), thereby demonstrating that enzyme constraints in central carbon metabolic pathways were also able to significantly reduce the flux variability in other metabolic subsystems.

### In silico metabolic engineering to increase γ-PGA flux

Poly-γ-glutamic acid is a biodegradable, water-soluble, non-toxic, and edible biopolymer that has a large number of biotechnological applications, ranging from biomedicine to bioremediation. For its unique proprieties, different studies for improving the microbial production of γ-PGA were carried out [52, 53]. The *pgs* operon, which includes the enzyme-coding genes responsible for the biosynthesis of this polymer, is not expressed in *B. subtilis* 168 laboratory strains. However, we previously demonstrated that *pgs* operon transcription could be activated in a *B. subtilis* 168 derivative by two mutations: *swrA*^+^ and *degU32*(Hy), thereby engineering γ-PGA production [15]. In this work, our goal was to use genome-scale models to identify the genetic perturbations (gene deletions) that could increase the production of γ-PGA.

**Fig. 6:**
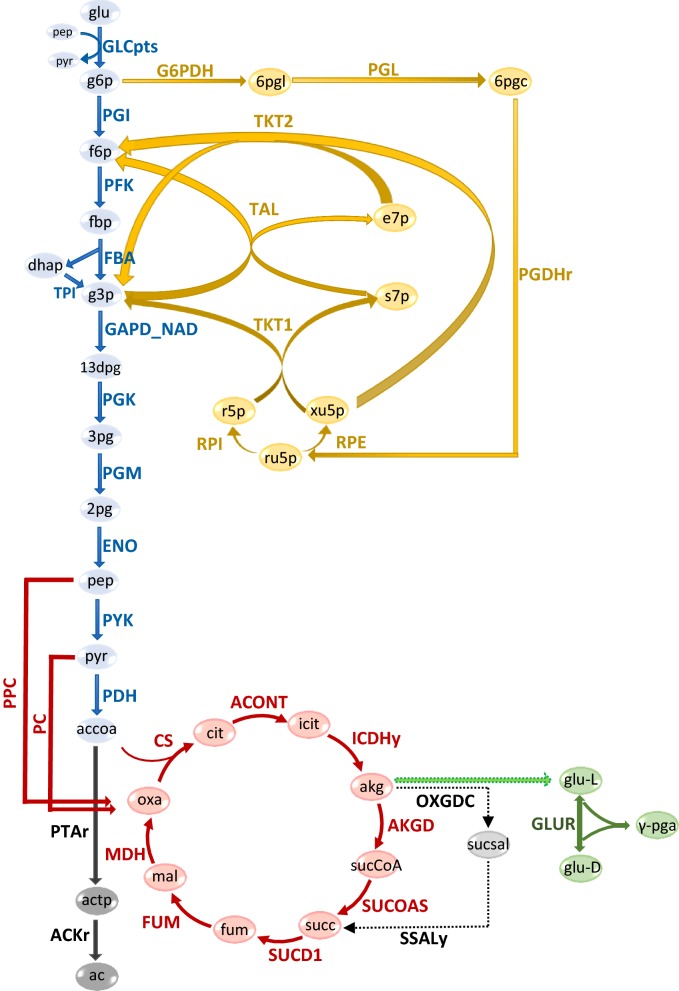
Central carbon and γ-PGA production pathways of *B. subtilis*. The central carbon pathway that includes the pathway of glycolysis (blue), the pentose phosphate pathway (yellow) and the TCA cycle (red) is represented. The pathway of acetate (dark gray) and γ-PGA (green) biosynthesis are also represented. Dashed thin arrows indicate the bypass pathway from akg to succ, with sucsal as intermediate metabolite. The green arrow with dashed edge indicates a set of reactions (not shown) converting akg into glu-L. Metabolite and reaction names are reported in lower and upper case, respectively, according to the annotation used in the original model

**Fig. 7:**
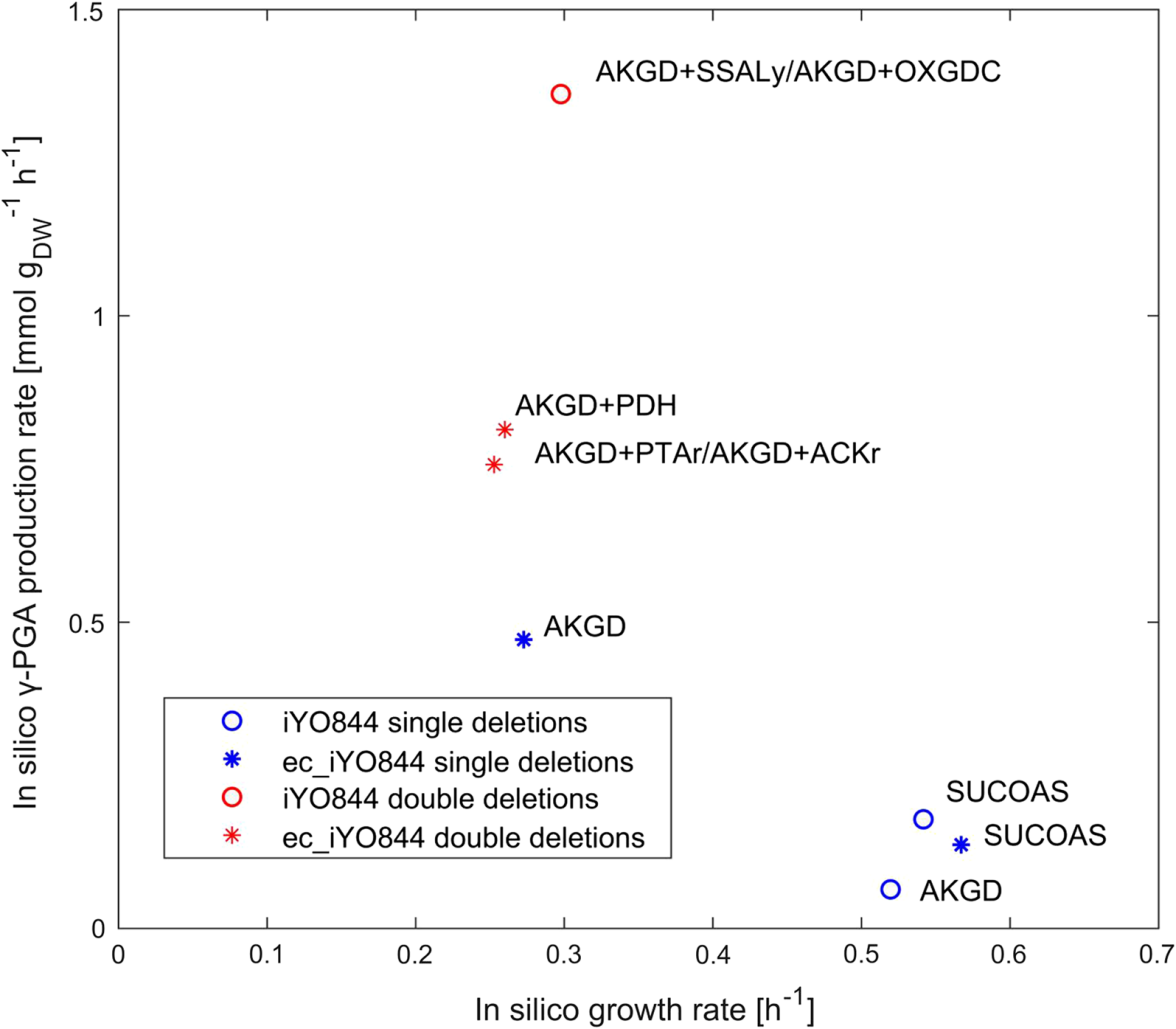
Reaction deletions predicted by MoMA to optimize the production of γ-PGA. Predicted γ-PGA flux and growth rate by iYO844 or ec_iYO844 for the single-reaction deletions and the best double-reaction deletions are reported. The slash indicates alternative double deletion solutions with the same predicted γ-PGA flux and growth rate. See text and Fig. 6 for a description of the reactions reported

We modified the iYO844 and ec_iYO844 models to include the γ-PGA biosynthesis reaction, and we used them to predict gene deletion targets for increasing flux through this reaction using the MoMA method (see “Methods”). Three γ-PGA precursors (akg, glu-L, glu-D) were also considered as target metabolites (see Fig. 6), but the deletion targets identified for these were the same as the ones identified for γ-PGA (data not shown). Both models predicted the 2-oxoglutarate dehydrogenase (AKGD) reaction (akg + coa + nad → CO_2_ + nadh + succoa) and the succinyl-CoA synthetase (SUCOAS) reaction (atp + coa + succ ↔ adp + pi + succoa) as the single-reaction deletion targets yielding the highest γ-PGA flux (see Fig. 7). Accordingly, a decrease of the proteins involved in the two target reactions was previously suggested to be beneficial to γ-PGA production [54], but no forward engineering evaluation of this strategy had previously been done.

Even if the same single-reaction deletion targets were identified by both models, and similar in silico γ-PGA flux and growth rate upon SUCOAS deletion were predicted by both models, the effects of AKGD deletion had a significant difference between the two models (see Fig. 7). ec_iYO844 predicted a fivefold higher γ-PGA flux than iYO844, along with a significant growth rate reduction. Using iYO844, the γ-PGA flux upon AKGD deletion was even lower than the one predicted with SUCOAS deletion.

Regarding the predicted flux distribution of the AKGD deletion mutant, a major difference between iYO844 and ec_iYO844 was that the former predicted the activation of the 2-oxoglutarate decarboxylase (OXGDC) and the succinate-semialdehyde dehydrogenase (SSALy) bypass reactions. While the SUCOAS and AKGD reactions are downstream of the akg production in the TCA cycle, OXGDC and SSALy (akg + h → co_2_ + sucsal, and h2o + nadp + sucsal → (2) h + nadph + succ, respectively) are consecutive reactions forming a bypass for the production of succinate (succ) from akg (see Fig. 6). Conversely, the ec_iYO844 model predicted this bypass reactions as inactive due to an enzymatic constraint on OXGDC (see Table 1), for which the associated protein (MenD) was not experimentally detected in these conditions [33, 55]. For this reason, when AKGD is deleted, ec_iYO844 predicted a higher flux from akg towards glutamate (and subsequently γ-PGA) than iYO844, in which akg was predicted to be mainly converted into succ and to continue the TCA cycle, with a lower flux towards glutamate. We confirmed that the bypass from akg to succ was the source of this key diversity between the models by simply imposing a null value of OXGDC in iYO844, as it was essentially done in ec_iYO844 by constraining the flux to very low levels. As expected, results predicted an increase of γ-PGA flux in the AKGD deletion strain (see Fig. 7).

Using the same procedure, we searched for double-reaction deletions to further compare the two models. As expected from the analysis above, for iYO844 the best double deletion found was the AKGD and OXGDC (or SSALy) combination (see Fig. 7). Using ec_iYO844, the three best deletions were the AKGD combination with the phosphotransacetylase (PTAr), acetate kinase (ACKr) or pyruvate dehydrogenase (PDH) reactions (see Fig. 7). The additional deletion of PTAr, ACKr or PDH predicted an increase of γ-PGA flux by decreasing the flux towards acetate (see Fig. 6), also consistent with a previous metabolic engineering work [56]. A similar conclusion could be drawn by considering the best double-reaction deletion target (AKGD + PDH) found using iYO844 with a null value for the OXGDC flux as additional constraint (data not shown). This target combination was immediately followed by combinations of AKGD with a set of PPP reactions, with lower γ-PGA flux (data not shown), not found using ec_iYO844, probably due to the major differences in the flux distribution of this pathway (as illustrated in Fig. 1). The results obtained by double-deletion analysis further confirmed that the flux through the OXGDC/SSALy bypass is a key difference between iYO844 and ec_iYO844, affecting the predicted deletion targets, but other enzymatic constraints can also play a role for specific strain design predictions.

In summary, the two models showed the same results in terms of recommended single-reaction deletion list (AKGD or SUCOAS), but the predicted fluxes showed quantitatively different values due to the constraints of the GECKO model. Such constraints also determine the difference between the two models in the suggested double-reaction deletion list, with iYO844 recommending the deletion of a reaction (OXGDC or SSALy) for which ec_iYO844 predicted a negligible flux.

### In vivo metabolic engineering to improve γ-PGA production

**Fig. 8:**
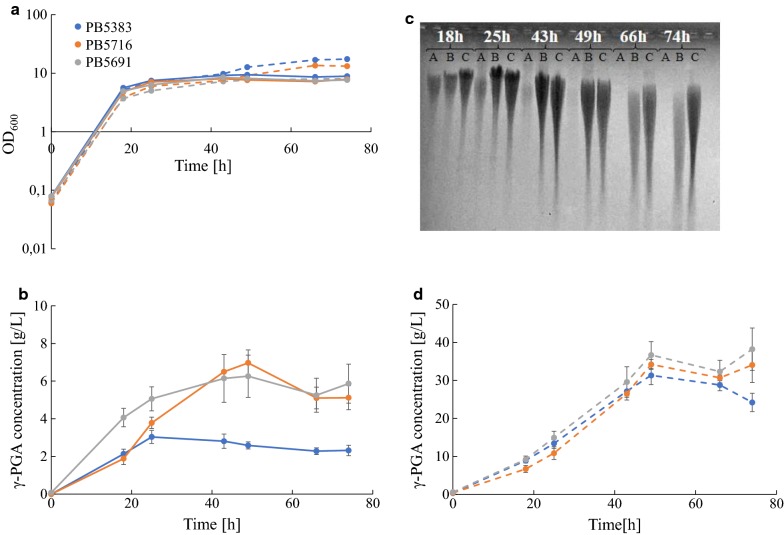
Fermentation experiments. **a** Growth curves in EM− and EM+ media. **b** γ-PGA concentration profiles in EM−. **c** Electrophoretic separation of γ-PGA collected in the experiments with EM−. **d** γ-PGA concentration profiles in EM−. The reference strain (PB5383) and the two mutants (PB5716 and PB5716) are indicated as A, B and C, respectively. In **a**, **b**, **d** circles represent average data points, error bars represent the 95% confidence intervals of the mean. Solid and dashed lines indicate experiments in EM− and EM+, respectively. In **c** the size distribution of the polymer can be qualitatively visualized. The intensity of the signal is comparable only among samples in which the polymer shows a similar distribution

The optimal single-reaction genetic configurations identified by in silico design were experimentally tested, comparing the γ-PGA biosynthesis performance of the PB5383 producer strain [15], used as reference, with two new isogenic strains in which the AKGD and SUCOAS reactions were disrupted. We limited our study to the single-reaction deletion list since it included targets that have never been tested in metabolic engineering studies. In *B. subtilis* the *odhA*–*odhB*–*pdhD* and *sucC*–*sucD* genes encode the two target reactions, respectively. Since the entire 2-oxoacid dehydrogenase multienzyme complex, encoded by *odhA*, *odhB* and *pdhD* genes, is necessary for the AKGD catalysis and the *pdhD* gene is also responsible for the pyruvate dehydrogenase (PDH) reaction, only *odhAB* and *sucCD*, both in operon architecture, were finally selected for in vivo deletion, giving rise to strains PB5691 and PB5716, respectively (see Table 2). We compared γ-PGA production between the three strains (PB5383, PB5691 and PB5716) in batch fermentation experiments in EM− medium. Results showed that the growth rate of the two knockout strains (0.47 h^−1^ and 0.33 h^−1^ for PB5691 and PB5716, respectively) was only slightly lower than PB5383 (0.54 h^−1^) and the growth profiles during fermentation were very similar among the three strains (solid lines Fig. 8a). In this condition, the reference strain was able to reach a maximum γ-PGA concentration of 3.1 g/L, at t = 24 h, with a subsequent decrease in polymer concentration (Fig. 8b), consistent with a previously reported profile obtained for *B. licheniformis* in a similar growth medium [57]. On the other hand, the two new knockout strains showed a very different γ-PGA concentration profile, with a significantly higher maximum concentration (p-value < 0.05; T-test), reached at t = 49 h (Fig. 8b), while the maximum concentrations reached by the two mutants (6.5 and 7.2 g/L for PB5691 and PB5716, respectively) were not significantly different from each other. The results were qualitatively confirmed by polymer separation on agarose gel electrophoresis (Fig. 8c): a higher concentration of γ-PGA was observed in PB5716 and PB5691 at different time points. As Fig. 8b suggests, biopolymer production rate also showed differences among the tested strains. In particular, the reference strain was characterized by a production rate per biomass unit (0.087 g/g_DW_/h) significantly lower than the knockout strains (p-value < 0.05; T-test). The production rate per biomass unit of PB5691 (0.18 g/g_DW_/h) was also significantly higher than the one of PB5716 (0.11 g/g_DW_/h) even if the two mutants reached a comparable maximum concentration of γ-PGA. In summary, the implementation of the identified knockouts yielded a twofold higher maximum concentration and per-cell production rate of γ-PGA, with the deletion of *odhAB* being the most promising one in terms of production rate per biomass unit, as predicted by the enzyme-constrained model.

The addition of 40 g/L of l-glutamate (EM+ medium), a direct precursor of γ-PGA, as in the original E medium composition, significantly improved biopolymer production compared to the EM− medium for all strains (Fig. 8d), while the growth profiles remained very similar both in presence and in the absence of glutamate (dashed lines Fig. 8a). The reference strain produced up to 32 g/L, while *odhAB* and *sucCD* mutants reached up to 43 and 38 g/L, respectively (not statistically significant difference compared to the reference strain; T-test). The per-cell production rate showed a significant difference between the reference strain (0.4 g/g_DW_/h) and the mutants, and between the two mutants (p-value < 0.05; T-test), with the *odhAB* deletion strain showing again higher production (0.7 g/g_DW_/h) than the *sucCD* mutant (0.43 g/g_DW_/h). The differences observed between fermentations in EM− and EM+ were expected, since l-glutamate is a direct precursor of γ-PGA; its presence results in increased γ-PGA flux, as previously measured [57]. For this reason, a smaller percent difference between the knockout and parent strains was also expected in EM+ than in EM−.

## Discussion

Following the principles of the recently proposed GECKO method, we integrated enzymatic and proteomic data for 17 central carbon-related pathways in an existing genome-scale metabolic model of *B. subtilis*. We showed that the resulting model, ec_iYO844, provides more accurate prediction of metabolic phenotypes compared to the original non-enzyme constrained model. Both secretion and intracellular flux predictions are considerably better with ec_iYO844 than iYO844 for wild-type and, to a lesser extent, mutant strains. Notably, the large errors in wild-type flux predictions observed in the original model, i.e. for the pentose phosphate pathway (in which no flux was predicted), acetate secretion and TCA cycle (which were under- and over-estimated, respectively), are all fixed in ec_iYO844. Moreover, the new model does not require to fix glucose uptake rate to an experimentally determined value, as was the case with the original model, since the glucose uptake flux is determined by enzyme capacity constraints. This advantage has been previously highlighted for other models adding constraints to existing genome-scale model [23]. A dataset of essential genes was also used to compare the accuracy of the initial and new model: the enzyme-constrained one provided a correct prediction (i.e., no growth) for more strains than the non-enzyme constrained model. The specific values retrieved for *k*_*cat*_ of enzymes are essential to achieve the improved prediction performance, since a randomized set of *k*_*cat*_ values could not provide a similar accuracy. However, not all the enzyme constraints for reactions were essential to reach the predictive accuracy of the ec_iYO844 model: constraints for only four (TPI, GAPD_NAD, CS and PGCDr) of the 17 reactions were sufficient under the conditions that we used in this study. It is important to highlight that this result is expected to be specific for the data used to evaluate predictions.

While the originally proposed GECKO method adopted a genome-scale constraining of reactions in yeast, in this study only 17 reactions involved in central carbon metabolism were enzyme-constrained based on manually curated enzyme kinetic and proteomic data. This small-scale constraining was sufficient to demonstrate the potential of this approach with the experimental data used, but larger-scale constraining would be expected to provide benefits for the prediction of reactions and metabolic engineering targets outside of central carbon metabolism. The limitations of small-scale constraining were apparent in the gene essentiality predictions for genes outside of central carbon metabolism, which were not affected by enzyme constraints for central carbon metabolic reactions. The primary challenge for developing constraints for reactions outside of central carbon metabolism is the limited availability of enzyme kinetic data for even relatively well-characterized organisms like *B. subtilis*. The poor availability of high quality enzyme kinetic data has been identified as a major impediment for developing improved computational models of cellular metabolism in general [58].

In contrast to essentiality predictions, enzyme constraints showed significant effect in reducing flux variability in reactions outside of central carbon metabolism. Out of the reactions with non-zero variability in the original iYO844 model, 81% of the reactions showed reduced flux variability. This demonstrates that enzymatic constraints can have an impact on different pathways apart from the one in which constrained are added. The result obtained for ec_iYO844 is consistent with the improvements observed in yeast with genome-scale enzyme constraining. The reduction in flux variability suggests that the ec_iYO844 model could be better for strain design than the original iYO844 model even for products that do not directly come from central carbon metabolism.

The evaluation of the models in a metabolic engineering study enabled the identification of two promising gene deletion targets in TCA cycle that led to the improvement in the production of γ-PGA, a biopolymer with a large number of applications. The model without and with the integration of enzymatic data predicted the same single-reaction deletion targets (AKGD and SUCOAS). However, differences were observed in the quantitative prediction of γ-PGA flux of mutant strains and in the list of recommended double-reaction targets. Among these, a remarkable difference was that the original model led to the selection of AKGD with OXGDC/SSALy as best deletion combination, while the enzyme-constrained model predicted that OXGDC/SSALy do not carry significant flux due to an undetectable protein level for one of the corresponding enzymes. Therefore, the strain design based on the original model was unnecessarily complex since the same result could be reached with a single-reaction knockout (AKGD).

Considering the single-reaction deletion targets, the inverse correlation of γ-PGA production with the level of the enzymes catalyzing SUCOAS and AKGD was previously observed by Yu et al. [54] in *B. licheniformis*, but, to our knowledge, no forward engineering studies have been done to confirm the effect of these knockouts. For this reason, the AKGD and SUCOAS reactions (encoded by *odhAB* and *sucCD* operons, respectively) were selected as deletion candidates in this study to test their effect on γ-PGA biosynthesis.

The two knockout strains (Δ*odhAB* and Δ*sucCD*) were constructed in vivo by implementing gene deletions in a previously engineered strain (PB5383), capable of γ-PGA production. Fermentation experiments showed that both mutants could reach a significantly higher (twofold) maximum γ-PGA concentration than PB5383 over a three-day growth in E medium without glutamate. The Δ*odhAB* strain also significantly outperforms the others in terms of per-cell γ-PGA production rate, with a two-fold improvement over PB5383. These results demonstrated that TCA cycle flux was successfully diverted towards glutamate, and subsequently toward γ-PGA production, by implementing the knockouts recommended by the model. As expected, the addition of l-glutamate in the medium increased γ-PGA production in all strains, since l- and d-glutamate are precursors of γ-PGA, but the Δ*odhAB* strain still showed improved production compared with the other strains.

## Conclusions

In summary, we have developed a *B. subtilis* genome-scale metabolic model with significantly improved ability to predict metabolic flux distributions and phenotypes. The improved model relies only on a limited set of enzymatic constraints derived from measured protein expression profiles and kinetic parameters reported in the scientific literature. The application of this model to predicting metabolic phenotypes and metabolic engineering designs demonstrated the utility of the model and the value of relatively limited enzymatic constraints. This work paves the way for improving genome-scale metabolic models of other industrially attractive organisms by incorporating enzyme kinetic and proteomic data available in databases and the scientific literature.
